# Cholesterol Induces Oxidative Stress, Mitochondrial Damage and Death in Hepatic Stellate Cells to Mitigate Liver Fibrosis in Mice Model of NASH

**DOI:** 10.3390/antiox11030536

**Published:** 2022-03-11

**Authors:** Einat Rauchbach, Haim Zeigerman, Diana Abu-Halaka, Oren Tirosh

**Affiliations:** Institute of Biochemistry, Food Science and Nutrition, The RH Smith Faculty of Agriculture, Food and Environment, The Hebrew University of Jerusalem, Rechovot 7610001, Israel; einat.rauchbach@mail.huji.ac.il (E.R.); haimz@savion.huji.ac.il (H.Z.); diana.abuhalaka@mail.huji.ac.il (D.A.-H.)

**Keywords:** apoptosis, lipid peroxidation, liver disease, fibrosis

## Abstract

Liver fibrosis and its end-stage disease cirrhosis are major world health problems arising from chronic injury of the liver. In recent years, the hypothesis that hepatic stellate cells’ (HSCs’) activation and fibrosis can be mitigated by HSC apoptosis and cell death has become of interest. In the current study, we evaluated the effect of cholesterol and bile acids on HSC apoptosis and liver fibrosis. Male C57BL/6J mice (wild type), aged four to five weeks, were fed an AIN-93G based diet (normal diet, ND), ND diet + 1% (*w*/*w*) cholesterol (CHOL group), ND diet + 0.5% (*w*/*w*) cholic acid (CA group) or ND diet + 1% (*w*/*w*) cholesterol + 0.5% (*w*/*w*) cholic acid (CHOL + CA group). Female Mdr2(-/-) mice were also treated with ND with and without 1% cholesterol. The effect of cholesterol on liver fibrosis and HSC clearance was evaluated. In addition, we studied the mechanism of cholesterol-induced apoptosis in HSC-T6 and AML-12 hepatocyte cell lines. In animals treated with cholic acids, increased lipid peroxidation and fibrosis were observed after six weeks of treatment. However, addition of cholesterol to the diet of C57BL/6J mice led to HSC-specific apoptosis and resolution of liver fibrosis, verified by double-staining with active caspase and α smooth muscle actin antibodies. In Mdr2 (-/-) mice, a diet supplemented with cholesterol corrected fibrosis and induced active hepatic stellate cells’ clearance. HSC-T6 were found to be much more sensitive to cholesterol-induced oxidative stress, mitochondrial damage and apoptosis compared to hepatocytes. These results indicate that cholesterol may be a trigger of HSC lipid peroxidation and death in the liver in a model of non-alcoholic steatohepatitis. A high cholesterol-to-bile acid ratio may determine the trajectory of the liver disease toward mitigation of fibrosis.

## 1. Introduction

Liver fibrosis and its end-stage disease cirrhosis are major world health problems arising from chronic injury of the liver. In recent years, the hypothesis that hepatic stellate cells’ (HSCs’) activation and fibrosis can be mitigated by clearance due to apoptosis and cell death has become of interest. Studies in rodents have demonstrated that experimental augmentation of HSC apoptosis will promote the resolution of fibrosis. Loss of activated HSC resulted from an increase in the rate of apoptosis during the first two days post-biliodigestive anastomosis, showing that HSC apoptosis plays a critical role in the spontaneous recovery from biliary fibrosis [1]. Moreover, activation of HSC ferroptosis (a necrotic type of cell death) was reported to mitigate hepatic fibrosis [2]. Consequently, there is now considerable interest in determining the molecular events that regulate HSC clearance [3].

The progression of NAFLD (nonalcoholic fatty liver disease) to NASH (nonalcoholic steatohepatitis) and then to hepatic cirrhosis and fibrosis is strongly influenced by the toxic effect of lipids, such as cholesterol, and bile acids [4]. The connection of free cholesterol (FC) to fibrosis in NAFLD is not clear since it may exacerbate pro-fibrotic treatments [5], but cholesterol can also promote hepatocytes’ proliferation to mitigate liver scarring when given in combination with bile acids or to MDR2 knockout (KO) female mice [6].

Cholesterol accumulation in the mitochondrial membrane of hepatocytes leads to depletion of reduced mitochondrial glutathione (mGSH) by disrupting the function of its carrier [7]. Mitochondrial cholesterol loading in hepatocytes impairs GSH transport and depletes mGSH stores, promoting mitochondrial ROS generation and lipid peroxidation [8]. Cholesterol is a pro-inflammatory molecule that can induce hepatocytes’ proliferation. Increased reactive oxygen species (ROS) production promotes the formation of lipid structures [9] by degrading hepatocytes with the potential to activate Kupffer cells and induce NF-κB’s (nuclear factor kappa-light-chain-enhancer of activated B cells’) nuclear translocation [10,11].

NASH’s histological features were correlated with significant alteration in bile acid composition, and cholestasis syndrome was detected in 3%, 34% and 47% of patients with NAFLD, NASH and liver cirrhosis, respectively [12]. Interestingly, while cholesterol was found to be highly associated with the induction of a liver inflammatory response [6,13], bile acids induce the expression of genes involved in extracellular matrix deposition in hepatic fibrosis and HSC activation, which are the main source of extracellular matrix (ECM) in liver fibrosis [13,14,15].

We hypothesize that toxic damage to the liver and fibrosis induced by bile acids can be mitigated by HSC elimination due to the cholesterol load. The current study evaluates the effect of cholesterol and bile acid in vivo and in vitro on HSC compared to hepatocytes. We demonstrate that cholesterol is specifically more toxic to HSC and can prevent bile acid-induced liver fibrosis.

## 2. Materials and Methods

Dietary-induced NAFLD study. Male C57BL/6J mice (wild type), aged four to five weeks, were purchased from Harlan Laboratories (Jerusalem, Israel). Wild-type mice (*n* = 40) were randomly divided into four experimental groups: mice fed standard AIN-93G diet [16] (normal diet, ND group), mice fed ND diet + 1% (*w*/*w*) cholesterol (CHOL group), mice fed ND diet + 0.5% (*w*/*w*) cholic acid (CA group) and mice fed ND diet + 1% (*w*/*w*) cholesterol + 0.5% (*w*/*w*) cholic acid (CHOL + CA group). The compositions of the diets are provided in Supporting Table S2.

Fibrosis study. In our Mdr2-/- cholesterol supplementation study, a multi-drug-resistance 2 knockout mice model (Mdr2-/-) was kindly provided by Professor Eitan Galun’s laboratory (Ein-Kerem, The Hebrew University of Jerusalem). In the present study, Mdr2-/- female mice were used because female mice display a more severe pathology of the disease than male mice [17]. Mdr2-/- mice, aged six to seven weeks, (*n* = 14) were randomly divided into two experimental groups: mice fed standard AIN-93G diet (Mdr2-/- normal diet, ND group) and mice fed the ND diet + 1% (*w*/*w*) cholesterol (Mdr2-/- CHOL group). During the experiment, all mice were housed in a temperature-controlled environment (20–24 °C), with a 12-h light/dark cycle and ad libitum access to food and water. Their body weights and food consumption were recorded weekly.

At the end of the experimental periods, the mice were subjected to overnight fasting and sacrificed randomly. Blood samples were collected in serum separator tubes and centrifuged (7155× *g* for 10 min at 4 °C) to obtain serum. Liver, epididymal fat tissue and blood samples were collected and stored at −80 °C until use. All procedures were performed according to the Institutional Animal Care and Use Committee (IACUC) of the Hebrew University of Jerusalem, ethics number AG-19-15986-3.

Liver histology, immunohistochemical studies and liver fibrosis staining. The tissues were fixed in 4% formalin and embedded in paraffin molds. Serial sections (5 μm thick) were cut from each block. One section of each block was stained with hematoxylin-eosin (H&E) for histological assessment, following standard procedures.

Liver fibrosis staining was performed using Trichrome Stain, according to the manufacturer’s instructions, using a commercial kit (TRM-500 by Scytek, West Logan, UT, USA). Immunohistochemical staining was performed on 4-µm sections using a Leica BOND-MAX system (Leica Biosystems Newcastle Ltd., Newcastle upon Tyne, UK). Slides were heated for 30 min at 60 °C, dewaxed and pretreated with an epitope-retrieval solution at pH 6 (ER1, Leica Biosystems Newcastle Ltd., Newcastle upon Tyne, UK), followed by incubation with anti-mouse alpha-smooth muscle actin primary antibody (NBP2-33006 by Novus bio) at a 1:1600 dilution for 30 min. Detection was performed with an alkaline-phosphatase detection kit (DS9390 by Leica Biosystems Newcastle Ltd., Newcastle upon Tyne, UK) and hematoxylin was used for counterstaining. Sequential double-immunohistochemical staining was performed using the Leica BOND-MAX system (Leica Biosystems Newcastle Ltd., Newcastle upon Tyne, UK). Tissues were pretreated with an epitope-retrieval solution at pH 6 (ER1, Leica Biosystems Newcastle Ltd., Newcastle upon Tyne, UK) for 10 min, followed by 30 min incubation with anti-cleaved caspase-3 antibody (1:1000, Abcam ab184787) and anti-alpha-smooth muscle actin (1:1600, NBP2-33006 by Novus Bio) primary antibodies. The Polymer Refine-HRP (cat. DS9800) and Refine-Red (cat. DS9390) kits, both from Leica Biosystems, were used for detection, and hematoxylin was used for counterstaining (liver fibrosis and immunohistochemical staining performed by Gavish Research Services, Ness Ziona).

Biochemical analysis of blood and serum parameters. The liver enzymes, serum alanine aminotransferase (ALT/SGPT) and serum aspartate aminotransferase (AST/SGOT) levels were measured by an automated clinical chemistry analyzer along with the total cholesterol, triglycerides and bilirubin (American Laboratories Ltd., Herzliya, Israel). Fasting glucose levels were measured in tail-tip blood with a handheld Optimum Xceed glucometer (Abbott Diagnostic Care Ltd., Oxon, UK).

Free cholesterol quantification. Free cholesterol (FC) was extracted as described by Paulazo and Sodero [18]. Briefly, mouse liver tissue (50 mg) or cell pellets of cultured HSC-T6 and AML-12, were homogenized in 800 µL of acid saline solution (ASS; NaCl—154 mM, HCl—15 mM). Following homogenization, 2 mL of methanol/1 mL of chloroform was added and vortexed for 20 s. Then, 1 mL of chloroform/1 mL of ASS was added, samples were vortexed, incubated on ice for 10 min and centrifuged for 5 min at 200× *g*. The upper phase was discarded, and the lower organic phase was mixed with 2 mL of methanol/1.8 mL of ASS. The samples were vortexed, incubated on ice for 10 min and centrifuged for 5 min at 200× *g*. Finally, the lower organic phase was collected and then 1 mL of the collected organic phase was evaporated under a nitrogen flow and resuspended in 1 mL of HPLC-grade isopropanol.

Following the extraction of FC, isocratic HPLC analysis was performed as previously described by Czauderna et al. [19], utilizing a Merck/Hitachi LaChrom HPLC system (diode array detector L-7455, pump L-7100 and programmable autosampler L-7250). The system was operated with a data processor working with a LaChrom interface D-7000 and the EZChrom Elite 3.3.1 software. The samples were separated on a Kinetex EVO C18 100 Å column (250 × 4.6 mm, 5 μm), utilizing the acetonitrile/isopropanol (87:17 *v*/*v*) mobile phase at the constant flow rate of 1.5 mL/min. The injection loop volume was 20 μL. The cholesterol peak was identified at 205 nm based on the retention time and UV spectrum of the standard cholesterol solutions.

Lipid peroxidation analysis. Liver lipid peroxidation was measured using a TBARS (thiobarbituric acid reactive substances) assay [20]. Liver tissue (50 mg) or cultured HSC-T6 and AML-12 was homogenized with 1 mL of 12% (*v*/*v*) TCA (trichloroacetic acid) and centrifuged at 14,841× *g* (Thermo Scientific™, Heraeus Megafuje 16R, Osterode am Harz, Germany) for 15 min. Fatty supernatant was gently removed and 500 µL of the soluble phase was separated and mixed with 500 µL of 1 mM TBA (thiobarbituric acid). The samples were incubated for 30 min at 100 °C. HPLC separation was performed on a Merck/Hitachi HPLC system, LaChrom L 7100 pump and LaChrom Fluorescence Varian ProStar 363 detector. The mixture was separated on a Kinetex EVO C18 column (250 × 4.6 mm, 5 μm) using 20 mM K_2_HPO_4_ buffer and 35% methanol as a mobile phase. A standard curve was obtained using 1,1,3,3 tetramethoxypropane as the malondialdehyde (MDA) standard. The flow rate was constant at 0.6 mL/min; the injection loop volume was 20 μL and was monitored at Ex 500/Em 560 nm. The results were normalized to the tissue weight.

Caspase-3 enzyme activity. The liver caspase-3 enzyme activity was determined with the caspase-3 substrate according to the manufacturer’s instructions (Caspase-3 Substrate II, Fluorogenic, Merck). In brief, liver tissue (20 mg) was homogenized in 1 mL Lysis Buffer (provided in Supporting Table S4) without a protease inhibitors cocktail and centrifuged at 19,746× *g* for 15 min. Fatty supernatant was removed and 500 µL of the soluble phase was separated and diluted with 500 µL of Lysis Buffer containing fresh DTT. For the assay, 100 µL from each sample was added to a black 96-well plate. Then, 7 µL of caspase-3 substrate was added to each well (20 µM final concentration), and the plate was incubated in the dark at 37 °C with shaking for 20 min. The total fluorescence was measured at Ex360/Em465 nm using an ELISA reader (Tecan, GENios) for 1.5 h every 10 min. The results were normalized to the initial tissue sample weight.

Cell line experiments. An immortalized HSC-T6 cell line of rat activated hepatic stellate cells (HSCs; generously provided by Dr. S.L. Friedman) was maintained at 37 °C in 5% CO_2_ in Dulbecco’s modified Eagle’s medium supplemented with 10% heat-inactivated fetal bovine serum (HI-FBS), 1 × glutamine and 1% PS (penicillin-streptomycin). After harvesting, cells were seeded at a concentration of 1.0 × 10^5^ cells/mL on a six-well plate. Approximately 24 h after seeding, HSC-T6 cells were washed twice with PBS and the medium was replaced with fresh serum-free medium with the following treatments, according to the experimental setting: control (only DMEM), vehicle (0.1% DMSO), CHOL (cholesterol, 0–500 µM) or CA (cholic acid, 200 µM).

Mouse AML-12 hepatocytes were obtained from ATCC (Israel). The AML-12 cells were grown in Dulbecco’s modified Eagle’s medium with high glucose, containing 10% fetal bovine serum (FBS) and 1% PS (penicillin-streptomycin) and maintained at 37 °C in a humidified environment with a 5% CO_2_/95% air atmosphere. After harvesting, the cells were seeded at a concentration of 1.2 × 10^5^ cells/mL in a six-well plate. Approximately 24 h after seeding, AML-12 cells were washed twice with PBS, and the medium was replaced with fresh medium with the following treatments, according to the experimental setting: control (only DMEM), vehicle (0.1% DMSO), CHOL (cholesterol, 0–500 µM; DMSO 0.1%) and CA (cholic acid, 200 µM; DMSO 0.1%). For this experiment, the vehicle group containing 0.1% DMSO was applied as a control.

Cell viability assay. Following treatment, the cell viability was detected as previously described [20]. Briefly, the loss of cell-membrane integrity was detected by using propidium iodide (PI) staining. Cells were trypsinized, washed with phosphate-buffered saline (PBS) and centrifuged 5 min for 1649× *g* (Thermo Scientific™, Heraeus Megafuje 16R) at 4 °C. Then, cells were resuspended in PBS. Cells were filtered through a 90-µm mesh grid and stained with PI (2 µg/mL). Data were collected from 10,000 cells, measured by flow cytometry (FACSort, BD) with the fluorescence setting of excitation at 488 nm and emission at 575 nm and analyzed using CellQuest software.

Mitochondrial membrane potential assay. The mitochondrial membrane potential (ΔΨm) was measured by flow cytometry, using the cationic and lipophilic dye tetramethylrhodamine ethyl ester (TMRE), which accumulates in the mitochondrial matrix. Decreased ΔΨm is indicated by a reduction in the TMRE-induced red fluorescence intensity. Following treatment, the cells were trypsinized, washed with PBS and incubated with TMRE in culture medium (75 nM; 15 min; 37 °C), prepared for flow cytometry and analyzed. Data were collected from 10,000 cells; the fluorescence was measured by flow cytometry (FACSort, BD) with red (FL3 channel) fluorescence recorded on a log scale and analyzed using CellQuest software.

Mitochondrial cytochrome C release. Following treatment, the cells were washed with room-temperature PBS, trypsinized and centrifuged at 1649× *g* (Thermo Scientific™, Heraeus Megafuje 16R) for 10 min at 4 °C. The supernatant was discarded and the pellet was resuspended in ice-cold PBS. Then, the cells were centrifuged again at 500× *g* for 10 min at 4 °C. The supernatant was discarded and the pellet was resuspended in 400 μL of ice-cold lysis buffer (provided in Supporting Table S5) and shacked at 4 °C. Then, the cells were centrifuged (2000× *g* for 10 min at 4 °C) and the supernatant was collected—this fraction contained the cytosolic proteins. Next, 400 μL of ice-cold lysis buffer B (provided in Supporting Table S6) was added to the pellet, resuspended by vortexing and incubated on ice for 30 min. After centrifugation (7000× *g* for 10 min at 4 °C), the supernatant was collected—this fraction contained the proteins from membrane-bound organelles (mitochondria, endoplasmic reticulum, Golgi, etc.) except those from the nucleus. The protein concentration from the cytosolic and membrane-bound organelles fraction was evaluated using the Bradford method. Lysates from each group were mixed with Sample Buffer × 6 (provided in Supporting Table S7) to produce final samples. Each sample of 50 µL contained 100 µg of protein and was loaded on acrylamide gel. The rest of the procedure was performed as described perilously (Western Blot analysis) using a primary antibody specific to cytochrome C (Santa Cruz Biotechnology), diluted 1:1000, and peroxidase-conjugated Affini-Pure goat anti-mouse IgG secondary antibody (Jackson Immuno Research Laboratories, Inc., West Grove, Pennsylvania 19390).

DNA fragmentation assay. Following treatment, DNA fragmentation in cells was detected. Cells were trypsinized, washed with PBS and fixed in 0.5 mL of 1% (*v*/*v*) paraformaldehyde (4% in PBS), on ice for 30 min. Then, cells were centrifuged for 5 min at 1649× *g* (Thermo Scientific™, Heraeus Megafuje 16R) and the supernatant was discarded. Cells were suspended in citrate buffer containing PI (provided in Supporting Table S8). Data were collected from 10,000 cells, fluorescence was measured by flow cytometry (FACSort, BD) with excitation at 488 nm and emission at 530 nm and we analyzed these using CellQuest software.

Statistical analysis. Values are presented as the mean ± SEM (for in vivo results) or mean ± SD (for in vitro results). Data were analyzed by unpaired two-tailed Student’s *t*-test or by analysis of variance (one-way ANOVA) followed by the Tukey-Kramer HSD post-hoc test utilizing the JMP 15 Pro software suites (SAS). The significance level was *p* < 0.05 for all analyses.

## 3. Results

### 3.1. Effect of Dietary Cholesterol and Cholic Acid on Hepatic Damage Markers

Mice were treated with cholic acid (CA), free cholesterol (CHOL) and both. Supplementary Figures S1–S4 indicate the effect of the diets on growth and metabolic parameters. Although food intake was the same in all groups, CA-treated groups (with or without cholesterol) had lower weight gain, which may indicate a toxic effect or increased energy expenditure (Figure S1). When cholesterol was added to bile acid, a dramatic increase in liver weight was observed (Figure S2), as was shown previously by us; this effect on the liver is due to massive proliferation of hepatocytes [6]. In parallel, a dramatic increase in inflammatory parameters was observed (Figure S3). These results correlated with serum cholesterol levels (Figure S4).

**Figure 1:**
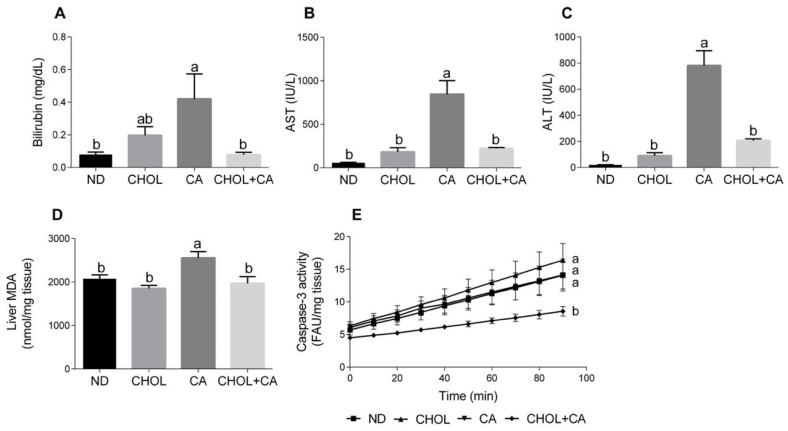
Effect of dietary cholesterol and cholic acid on hepatic damage markers. Male C57BL/6J mice aged four to five weeks were fed: ND—normal diet (*n* = 10), CHOL—cholesterol diet (*n* = 10), CA—cholic acid diet (*n* = 9), CHOL + CA—cholesterol and cholic acid diet (*n* = 8) for six weeks. (**A**) Serum bilirubin levels. (**B**) Serum AST levels. (**C**) Serum ALT levels. (**D**) Malondialdehyde (MDA) liver content. (**E**) Liver caspase-3 activity. All values are expressed as mean ± SEM. Columns indicated with different letters (a, b) are significantly different (*p* < 0.05) in Tukey-Kramer post-hoc test.

Several markers of liver injury were evaluated. As shown in Figure 1A, cholic acid supplementation (CA group) enhanced bilirubin levels in serum compared to other study groups (*p* < 0.05). Elevated serum levels of aspartate transaminase (AST) and alanine transaminase (ALT) are indicators of hepatic damage and hepatocyte destruction [21]. As shown in Figure 1B,C, compared to all other study groups (ND, CHOL, CHOL + CA) supplementation of CA alone significantly elevated the AST and ALT activity in the serum, respectively. There was no significant difference in liver enzyme levels between the ND, CHOL and CHOL + CA groups. Lipid peroxidation levels were measured by evaluating the malondialdehyde level (MDA) (Figure 1D). The CA-treated group showed significantly higher levels of lipid peroxidation, as indicated by the MDA in the liver, compared to all other treatments. Caspase-3 activity, an indicator of tissue apoptotic activity [22], was measured (Figure 1E). The ND, CHOL and CA groups showed no significant difference in caspase-3 activity, while the addition of cholesterol to cholic acid supplementation (CHOL + CA) reduced its activity significantly, indicating the average inhibition of apoptotic cell death in the whole liver mainly due to an increase in hepatocytes’ proliferation (these data are in correlation with an increase of liver size due to cholesterol supplementation, Figure S2).

### 3.2. Effect of Dietary Cholesterol and Cholic Acid Supplementation on Tissue Features and Fibrosis

**Figure 2:**
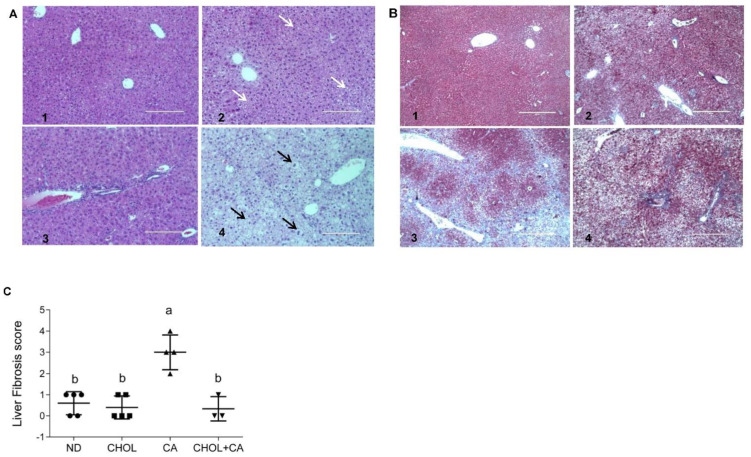
Effect of dietary cholesterol and cholic acid supplementation on tissue histology of features of fibrogenesis. Male C57BL/6J mice aged four to five weeks were fed: ND—normal diet, CHOL—cholesterol diet, CA—cholic acid diet, CHOL + CA—cholesterol and cholic acid diet for six weeks, with five animals in each treated group. (**A**) Representative liver H&E staining under ×20 magnification, scale bar 200 μm, for (**A1**) ND group; (**A2**) CHOL group (presence of microvascular fat accumulation marked with white arrows); (**A3**) CA group (presence of necrotic cells with malformed nuclei); (**A4**) CHOL + CA group (presence of ballooning degeneration of hepatocytes, accumulation of lipid droplets and Mallory-Denk bodies). Hepatocyte ballooning marked with black arrows. (**B**) Representative liver Masson’s trichrome staining under ×40 magnification, scale bar 400 μm, for (**B1**) ND group; (**B2**) CHOL group (few visible collagen lesions); (**B3**) CA group (high presence of fibrotic collagen lesions colored blue); (**B4**) CHOL + CA group (few visible collagen lesions). (**C**) Liver fibrosis score. Columns indicated with different letters (a, b) are significantly different (*p* < 0.05) in Tukey-Kramer post-hoc test.

Liver sections from all treated groups were stained (H&E). Figure 2A shows representative liver sections. In the ND (A1) and CHOL (A2) groups, a well-defined hepatocyte nuclei structure can be observed. On the other hand, in the CA (A3) and CHOL + CA (A4) groups, an abnormal hepatocytes histology was observed. The CA group (A3) presented multiple areas of malformed and shrunk nuclei of damaged hepatocytes, along with extended necrotic areas. The CHOL + CA (A4) group presented significantly increased cellular infiltration in the peri-portal area, some hepatocyte ballooning and the presence of Mallory-Denk hyaline. Masson’s trichrome staining was used to demonstrate connective tissue accumulation, mainly collagen formation, to evaluate liver fibrosis and scarring [23]. As presented in Figure 2B, the ND group (B1) showed no fibrotic areas. Cholesterol-supplemented groups CHOL (B2) and CHOL + CA (B4) showed minimal accumulation of fibrous collagen, while the CA (B3) group (supplementation with cholic acid) presented large blue-stained areas of collagen, indicating bridging fibrosis. We performed scoring evaluation by the following criteria: fibrosis was scored zero to four (zero, no fibrosis; one, periportal fibrosis; two, periportal fibrosis and fibrosis along the hepatic cord; three, bridging fibrosis; four, massive bridging fibrosis and cirrhosis). The results indicated an average value ± SEM of 0.6 ± 0.1 (*n* = 5) for ND, 0.4 ± 0.1 for the CHOL-treated group (*n* = 5), 3 ± 0.2 for the CA-treated group (*n* = 4) and 0.3 ± 0.2 for the CHOL + CA-treated group (*n* = 3), Figure 2C.

### 3.3. Effect of Dietary Cholesterol and Bile Acids on Pro-Fibrotic Genes

**Figure 3:**
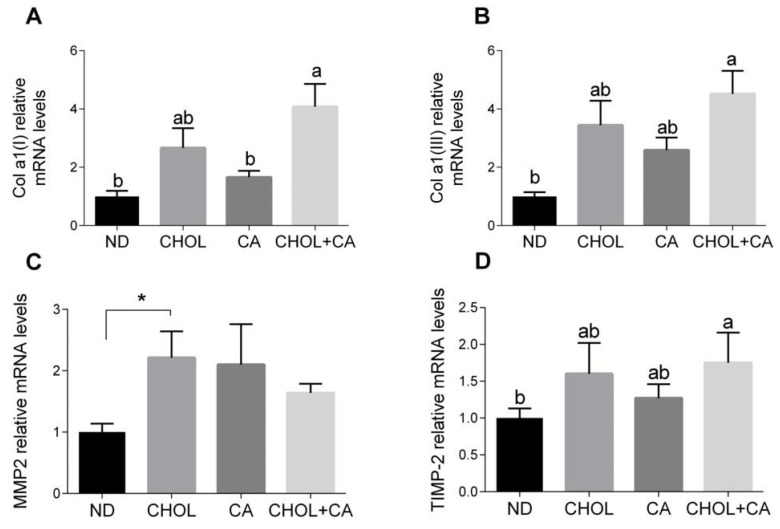
Effect of dietary cholesterol and bile acids on ECM genes. Male C57BL/6J mice aged four to five weeks were fed: ND—normal diet (*n* = 10), CHOL—cholesterol diet (*n* = 10), CA—cholic acid diet (*n* = 9), CHOL + CA—cholesterol + cholic acid diet (*n* = 8) for six weeks. (**A**) Col a1(l) relative mRNA levels. (**B**) Col a1(lll) relative mRNA levels. (**C**) MMP2 relative mRNA levels. (**D**) TIMP-2 relative mRNA levels. All values are expressed as mean ± SEM; columns marked with * are different from control according to Student’s *t*-test (*p* < 0.05); columns marked with different letters (a, b) are significantly different (*p* < 0.05) in Tukey-Kramer post-hoc test.

As shown in Figure 3A,B, the Col α1[I] (pro-collagen α1(I)) and Col α1[III] (pro-collagen α1(III)) mRNA levels were elevated significantly by CHOL treatment compared to treatments with ND and CA, respectively. MMP-2 is an autocrine proliferation and migration factor for HSCs [21]. As shown in Figure 3C, cholesterol supplementation (CHOL group) elevated the MMP-2 mRNA levels compared to ND (Student’s *t*-test, *p* < 0.05). The CA group also tended to show higher MMP-2 mRNA levels compared to ND (Student’s *t*-test, *p* < 0.052). Adding cholesterol to bile acid decreased the expression of MMP2. MMPs’ activity is regulated by TIMPs (tissue inhibitors of metalloproteinases). The addition of cholesterol to cholic acid supplementation (CHOL + CA group) showed significantly higher TIMP-2 mRNA levels compared to the ND group (Figure 3D). Altogether, statistically, there was little effect on ECM-regulating genes for all dietary treatments, indicating that the mechanism for controlling and mitigating fibrosis by cholesterol is probably a cellular survival effect.

### 3.4. Dietary Cholesterol and Cholic Acid Effect on Hepatic Stellate Cells’ Apoptosis

**Figure 4:**
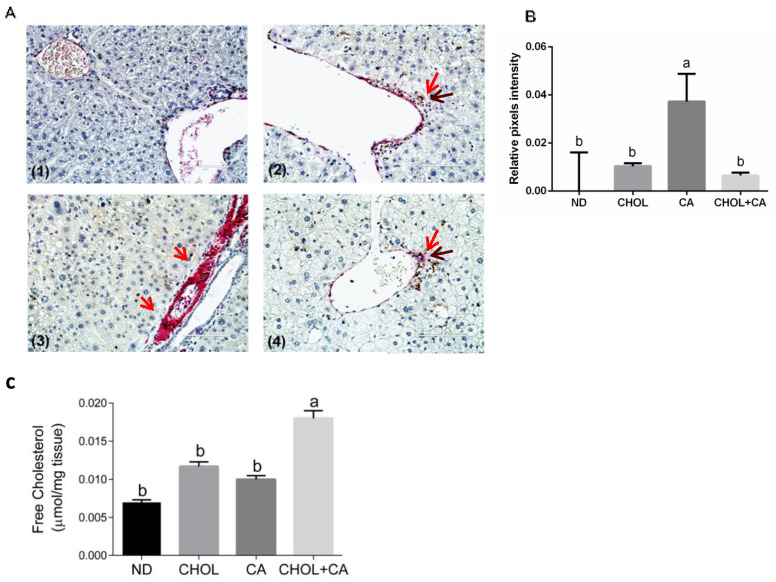
Dietary cholesterol and cholic acid effect on hepatic stellate cells’ apoptosis and liver tissue fibrosis. Male C57BL/6J mice aged four to five weeks were fed: ND—normal diet (*n* = 10), CHOL—cholesterol diet (*n* = 10), CA—cholic acid diet (*n* = 9), CHOL + CA—cholesterol and cholic acid diet (*n* = 8) for six weeks. (**A**) Representative histology of liver cleaved caspase-3 (brown) and αSMA (red) sequential double immunohistochemical staining, ×40 magnification, scale bar 100 µm; dietary-induced NAFLD models of (**1**) ND, (**2**) CHOL, (**3**) CA and (**4**) CHOL + CA, with five animals in each treated group. Red arrows represent αSMA stained cells; brown arrows represent cleaved caspase-3-stained cells. (**B**) Histological sections of three individuals from each group were imaged using the EVOS M500 microscope. Two frames from each slide were captured at a magnification of ×200. All captured images were at a resolution of 2048 × 1536 pixels and saved as TIF files. ImageJ software was employed to determine the relative intensity of pixels from the red channel (red/green + blue) of the total area of each image. For graphical presentation, the average value for the relative pixel intensity of the ND group was subtracted from those of all treatments. (**C**) Levels of free cholesterol in the liver. All values are expressed as mean ± SEM. Columns indicated with different letters (a, b) are significantly different (*p* < 0.05) in the Tukey-Kramer post-hoc test.

Recent proof-of-concept studies in rodents demonstrated that HSC apoptosis may promote fibrosis resolution [3]. We used caspase-3 (in brown) and αSMA (in red) sequential double immunohistochemical staining to evaluate this. As presented in Figure 4A, in the CA-treated group compared to ND, CHOL and CHOL + CA IHC, staining demonstrated a higher expression of α-SMA (in red) in the periportal region, a marker of activated HSCs. Apoptotic HSCs were detected in groups supplemented with cholesterol (CHOL and CHOL + CA) but not in the CA group. As shown in Figure 4B, αSMA staining intensity evaluation indicated that the CA group scored significantly higher than the rest of the treatments (*p* < 0.05). The free cholesterol levels were evaluated in the liver tissue (Figure 4C) and they correlated with the degree of apoptosis of tissue HSC. Using the genetic model of Mdr2 (-/-) in female mice, we demonstrated that adding cholesterol to the diet significantly ameliorated tissue fibrosis [6]. Therefore, we used this model to further investigate cholesterol’s effect on activated HSC using αSMA staining (Figure S5). As presented in Figure S5, cholesterol supplementation decreased α-SMA-positive cells’ presence in the liver tissue and significantly decreased α-SMA protein expression compared to the control group, indicating clearance of HSC from the liver tissue.

### 3.5. Effect of Cholesterol on HSC-T6 Cells’ Viability

**Figure 5:**
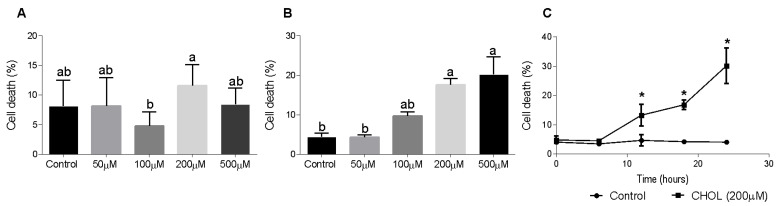
Effect of cholesterol on HSC-T6 viability. HSC-T6 cells were seeded in 96-well plates and exposed for 6 (**A**) and 24 (**B**) hours to cholesterol at four different concentrations (*n* = 4): 50 µM, 100 µM, 200 µM and 500 µM. The control group was treated with cell media only. (**C**) 200 µM cholesterol (CHOL (200 µM) ■) treatment group compared to the control group (Control ●) over consecutive time points (0–24 h). Cell viability was measured by applying a cell viability assay using PI staining, followed by measurement in flow cytometry. All values are expressed as the mean ± SD; columns marked with * are different from the control according to Student’s *t*-test (*p* < 0.05). Columns marked with different letters (a, b) are significantly different (*p* < 0.05) in the Tukey-Kramer post-hoc test.

To evaluate CHOL impact on HSC death, we used the HSC-T6 cell line. Dose-response and time-dependent experiments showed the cytotoxic effect of cholesterol on HSC-T6 cells. HSC-T6 cells were treated for 6 h with cholesterol at different concentrations (50–500 µM), as presented in Figure 5A. No significant cell death was observed at 6 h. Figure 5B shows cell-death induction in a dose-dependent manner in HSC-T6 cells treated for 24 h with cholesterol at different concentrations (50–500 µM). A time-dependent experiment was performed using 200 µM cholesterol treatment in HSC-T6 over consecutive time points (0–24 h). At the 12-h time point, the cells started to die from the treatment; there was a significant elevation in cell death in CHOL- treated cells compared to controls (Figure 5C).

### 3.6. Free Cholesterol (FC) Content in HCS-T6 and AML-12 Cells

**Figure 6:**
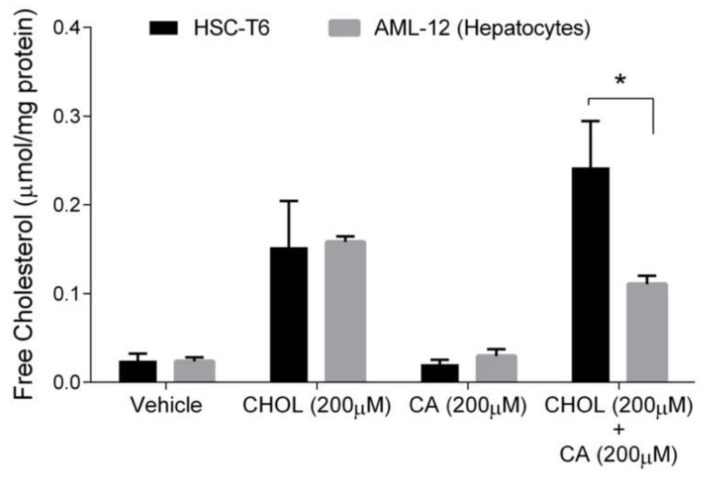
The effect of cholesterol and cholic acid on the free cholesterol (FC) content in HCS-T6 and AML-12 cells. HSC-T6 and AML-12 cells were seeded in 96-well plates and exposed for 24 h to the vehicle group (treated with 0.1% DMSO), cholesterol (CHOL 200 µM), cholic acid (CA 200 µM) or cholesterol with cholic acid (CHOL 200 µM + CA 200 µM), *n* = 4. All values are expressed as mean ± SD; columns marked with * are different from the vehicle according to Student’s *t*-test (*p* < 0.05).

Free cholesterol (FC) is considered the toxic form of cholesterol. Hepatocytes, unlike stellate cells, can convert cholesterol to bile acid to remove cholesterol excess. Therefore, we compared FC levels in hepatocytes and HSC. As shown in Figure 6, the FC content was significantly elevated under CHOL 200 µM and CHOL 200 µM + CA 200 µM treatments, compared to the vehicle and CA 200 µM treatments, in both HSC-T6 and AML-12 (*p* < 0.05 in Tukey-Kramer post-hoc test, not shown). As presented, no significant difference was observed between HSC-T6 and AML-12 under the following treatments: vehicle, CHOL 200 µM and CA 200µM. The FC content in HSC was twofold higher than in hepatocytes (AML-12) following treatment with CHOL 200µM + CA 200 µM.

### 3.7. Comparing the Effect of Cholesterol on HSC-T6 and AML-12 Cells’ Viability

**Figure 7:**
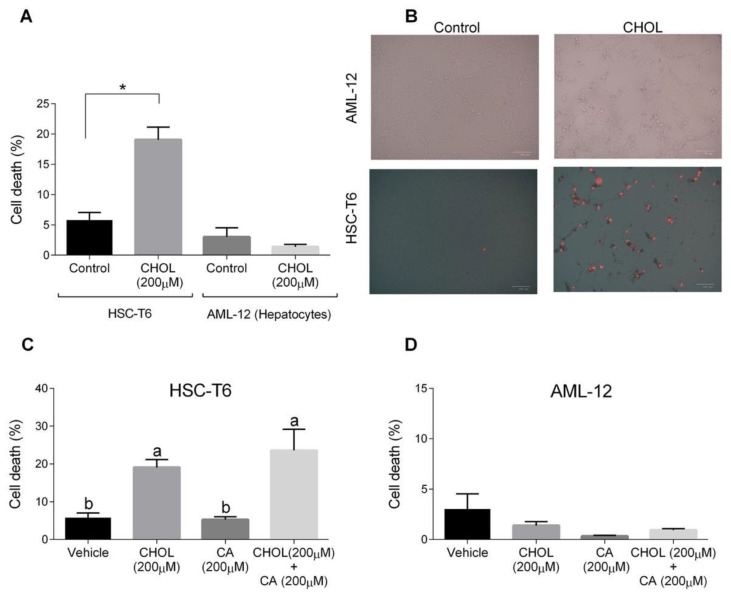
The effect of cholesterol and cholic acid on HSC-T6 and AML-12 viability. HSC-T6 and AML-12 cells were seeded in 96-well plates and exposed for 24 h to the control (treated only with cell media), vehicle group (treated with 0.1% DMSO), cholesterol (CHOL 200 µM), cholic acid (CA 200 µM) or cholesterol with cholic acid (CHOL 200 µM + CA 200 µM), *n* = 4. (**A**) Cell death in HSC-T6 and AML-12 cells. (**B**) Propidium iodide (PI) staining in HSC-T6 and AML-12 cells, following CHOL treatment; red spots indicate the loss of membrane integrity and cell death. (**C**) Cell death in HSC-T6 cells following CHOL and CA treatment. (**D**) Cell death in AML-12 cells following CHOL and CA treatment. All values are expressed as the mean ± SD; columns marked with different letters (a, b) are different (*p* < 0.05) in the Tukey-Kramer post-hoc test; columns marked with * are different from the control or vehicle according to Student’s *t*-test (*p* < 0.05).

The cytotoxic effects of both cholesterol and cholic acid in hepatocyte (AML-12) and HSC (HSC-T6) cell lines were evaluated. HSC-T6 cells treated with CHOL (200 μM cholesterol) had a significantly higher cell death percentage compared to the control group (Figure 7A). On the other hand, AML-12 hepatocytes treated with 200 μM cholesterol showed a low cell-death percentage, without any significant difference compared to the control group. On-plate staining with propidium iodide (PI) showed multiple positive PI-stained cells in the HSC-T6 treated with CHOL compared to the HSC-T6 control cells (Figure 7B). AML-12 control and CHOL-treated cells displayed negative responses to PI staining. In Figure 7C, HSC-T6 cells treated with CHOL (200 μM cholesterol) and CHOL + CA (200 μM cholesterol + 200 μM cholic acid) exhibited significantly higher cell death percentages compared to the control or only CA (200 μM cholic acid) treatment. As presented in Figure 7D, AML-12 cells treated with the vehicle, CHOL, CA or CHOL + CA exhibited a low percentage of cell death, as was evaluated by a PI exclusion assay and flow cytometry analysis.

### 3.8. Cholesterol Regulates Oxidative and Mitochondrial Stress in HSC, Facilitating Apoptosis

**Figure 8:**
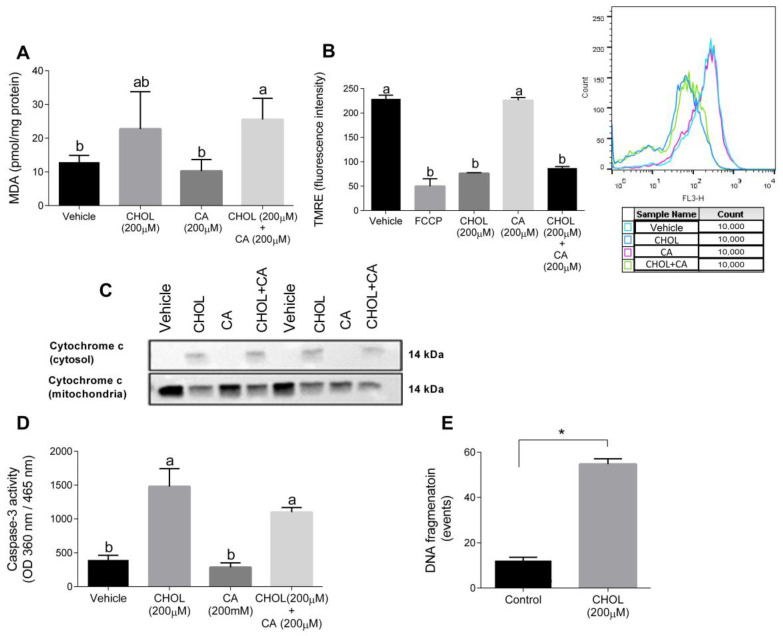
Cholesterol induces oxidative, mitochondrial stress and apoptosis in HSC-T6. HSC-T6 were exposed for 12–24 h to cholesterol (CHOL, 200 µM), cholic acid (CA, 200 µM), cholesterol with cholic acid (CHOL + CA, 200 µM) or the vehicle (treated with 0.1% DMSO). (**A**) HSC-T6 MDA cell content. (**B**) HSC-T6 mitochondrial membrane potential. (**C**) Cytochrome c leakage from mitochondria to cytosol 12 h post-treatment. (**D**) Caspase-3 activity 12 h post-treatment. (**E**) DNA fragmentation 24 h post-treatment. All values are expressed as the mean ± SD. Columns marked with * are different from control according to Student’s *t*-test. (*p* < 0.05), *n* = 4. Columns marked with different letters (a, b) are significantly different (*p* < 0.05) in the Tukey-Kramer post-hoc test. FCCP, potent uncoupler of mitochondrial oxidative phosphorylation.

Figure 8A shows that compared to the control, the MDA levels were significantly elevated in HSC-T6 treated with 200 µM cholesterol with or without 200 µM cholic acid. The mitochondrial membrane potential, detected using TMRE, declined with CHOL and CHOL + CA compared to the vehicle and CA groups (Figure 8B). We attempted to clarify the cell death mechanism induced by cholesterol treatment in HCT-T6. As shown in Figure 8C, following 12-h treatment (CHOL, CA or CHOL + CA), cytochrome-c leakage from the mitochondria was evaluated by the appearance in the cytosolic fraction and depletion in the mitochondrial fraction. Cytosolic leakage appeared in HSC-T6 cells treated with cholesterol (CHOL, CHOL + CA), but not after treatment with the vehicle or CA. After 12 h treatment, CHOL- and CHOL + CA-treated cells exhibited significantly higher caspase-3 activity compared to the vehicle and CA groups (Figure 8D). Further examination revealed that HSC-T6 cells treated with 200 µM cholesterol (CHOL) showed higher DNA fragmentation events compared to the vehicle group (*p* < 0.0001), following 24 h of treatment (Figure 8E). All of these indicate activation of the apoptotic pathway in HSC by FC.

## 4. Discussion

Apoptosis of HSC was suggested to be a mechanism for regression and prevention of liver fibrosis. It was suggested that these cells are resistant to TNFα-induced apoptosis due to BCL-2 (B-cell lymphoma 2) expression [21]. Clearance of HSC by apoptosis will promote the resolution of fibrosis, and there is an attempt to discover compounds and pharmacological agents that can induce HSC death [3]. Here, we showed that cholesterol, if brought to the liver at high enough concentrations by bile acids, is a selective oxidative stress inducer and proapoptotic compound in HSC. NAFLD progression is strongly influenced by the toxic effect of lipids and bile acids. Bile acids’ toxicity is an essential factor in metabolic disorders such as NAFLD and NASH. Furthermore, alternation in bile acids’ composition in the liver promotes activation of inflammatory, oxidative stress and necrotic cell death pathways and promotes liver fibrosis by activating HSCs [14,24,25]. In humans, NASH’s histological features were correlated with significant elevation and alteration in bile acid composition [12]. The molecular mechanisms by which bile acids initiate liver injury include direct detergent cytolytic effects and an indirect effect caused by elevation in ER stress and mitochondrial damage [26]. Our study demonstrated that cholesterol supplementation can induce oxidative stress in HSC and can lead to mitochondrial dysfunction and apoptosis. In fact, cholesterol can ameliorate bile acid-induced hepatic injury, although it is a pro-inflammatory molecule that can activate pro-inflammatory signaling within hepatocytes [27]. Cholic acid supplementation (CA group) enhanced bilirubin levels in the serum, and the addition of cholesterol (CHOL + CA) significantly reduced its levels, indicating a possible protective effect from bile hepatotoxicity. The CA group presented a dramatic elevation in the examined liver enzymes (ALT and AST), indicating enhanced liver damage in hepatocytes. Alternatively, cholesterol addition to the cholic acid supplemented group (CHOL + CA) significantly reduced liver enzyme levels in the serum. Resulting from treatment with dietary cholic acid, liver fibrosis was observed within a few weeks of treatment in WT mice. In Mdr2(-/-) female mice, the same happened spontaneously (with aging). Addition of cholesterol to the bile acid treatment or to the Mdr2 (-/-) diet significantly attenuated liver fibrosis. These results are in correlation with the clearance of activated HSC, as was evaluated by αSMA staining. An additional parameter indicating cell death is the activation of caspase-3; its activity was examined to assess the cholic acid and cholesterol effects on whole-liver homogenates (mainly hepatocytes). Caspase-3 activity was not elevated following the dietary treatments and was downregulated in the CHOL + CA group of mice (the group with the highest cholesterol levels in the liver). Indeed, there is growing evidence for an anti-apoptotic effect of cholesterol in hepatocellular carcinoma (HCC) cells [28]. In addition, there is a pro-proliferative and survival effect of cholesterol in the whole liver.

As presented in our work (Figure 2A,B) and by others, histological evaluation showed that the cholic acid-supplemented group (CA group) exhibited necrotic lesions, multiple necrotic nuclei and abnormal hepatic triads structures, emphasizing the toxicity of bile elevation to liver tissue [26,29]. In comparison, cholesterol addition (CHOL + CA group) led to a significant manifestation of inflammation with fewer hepatocytes’ necrosis and liver fibrosis. Double IHC staining of α-SMA and active caspase-3 revealed a cholesterol-mediated apoptotic effect specifically in HSC. A high prevalence of apoptotic HSC in cholesterol-supplemented groups (CHOL and CHOL + CA) was observed, while cholic acid supplementation (CA group) demonstrated a high prevalence of positive α-SMA cells (HSC). Massive elimination of activated HSC was also observed in the Mdr2(-/-) mice after supplementation with cholesterol (Supplementary Figure S5).

Recently, it was demonstrated in a cross-sectional study that the total bile acids (TBA)/to total cholesterol (TC) ratio could serve as a marker for liver fibrosis and cirrhosis in hepatitis B-infected patients [30]. The ratio of serum total bile acids to cholesterol reflects how bile acid levels are higher in cirrhosis while TC is significantly lower. The accumulation of serum BAs was also reported to be the main characteristic of cholestatic liver diseases that lead to hepatocyte death as well as progress of fibrosis [31]. It is clear that the increase of serum bile acid levels reflects the increase in hepatocytes’ bile acids content and leakage via the basolateral side of the hepatocytes to the circulation through the multidrug resistance-associated gene MRP4 (multidrug resistance protein 4) system [30]. In addition, it is known that cell extracellular matrix interactions can regulate growth and differentiation of hepatocytes. Modification of their interaction possibly stimulates hepatocyte proliferation, to directly mitigate fibrosis. This could be used as a treatment strategy since during hepatocyte proliferation, fibrosis is significantly mitigated [32]. In that context, cholesterol is a crucial factor for hepatocyte proliferation during liver regeneration [33]. We demonstrated that cholesterol may induce intrinsic hepatocyte proliferation via the Nrf-2 (nuclear factor-erythroid factor 2-related factor 2) system, meaning it can, therefore, mitigate liver fibrosis [6]. It has been reported, however, that cholesterol may exacerbate fibrosis in the presence of toxic treatments such as CCl4 (carbon tetrachloride) and TAA (thioacetamide) administration [34]. Therefore, the protective effect of cholesterol to mitigate bile acid-induced fibrosis still needs to be further validated. In addition, the current study indicates for the first time a direct proapoptotic effect of cholesterol on stellate cells.

We attempted to shed light on the mechanism by which cholesterol can facilitate cell death in HSC. Lipid peroxidation rates were evaluated for tissue damage and were assessed by measuring the malondialdehyde (MDA) levels [35]. The cholic acid load in the diet led to enhanced MDA production compared to the ND and CHOL groups. Interestingly, excessive MDA production was lowered significantly by cholesterol addition (CHOL + CA), Figure 1D. Cholesterol is an essential structural component of mammals’ cell membrane, where it regulates the lipid bilayer dynamics and structure and is required for normal cell function. The accumulation of cholesterol in cell membranes might lead to a more rigid membrane structure and increased mitochondrial oxidative damage in the whole liver on average [36].

The data indicate that cholesterol can shift the trajectory of liver disease from advanced fibrosis to more proliferation of hepatocytes and elimination of HCS, which may lead in the long run, to more inflammation and hepatocellular carcinoma (without necessarily causing cirrhosis). Therefore, loading the liver with cholesterol can be considered (if at all) only for a short time, to trigger liver regeneration and elimination of HSC and fibrosis.

Emerging experimental and clinical evidence is starting to show that fibrosis and cirrhosis are potentially reversible in humans and rodents [3,36,37,38]. α-SMA IHC staining showed that HSC is highly prevalent and activated under CA supplementation, while cholesterol (CHOL + CA) decreased its prevalence. In Mdr2-/- cholesterol supplementation, a significant reduction in tissue fibrous collagen was shown [39]. In our study, further examination revealed lower levels of HSC-positive cells and lower protein expression of α-SMA by western blot analysis. In periportal regions of the liver, HSC apoptosis was demonstrated by caspase-3 staining. To clarify the mechanism by which cholesterol may cause apoptosis in HSC, we used the HSC-T6 cell line. Cholesterol was shown to possess a cytotoxic effect in the HSC-T6 cell line with a dose-response and time-dependent manner. Cholesterol downregulates genes involved with HSC activation, survival and fibrogenesis, such as α-SMA, Col α1[I], c-Myc and MMP-2 (not shown). Examination of FC accumulation in HSC-T6 and AML-12 cells treated with cholic acid and cholesterol showed that both cell lines significantly increased their FC content in the presence of cholesterol in the media. However, when treated with cholic acid and cholesterol together, there was a significantly higher FC accumulation in HSC-T6 than in AML-12 hepatocytes. The presence of cholic acid allowed better solubilization of cholesterol and thus mediated its increased levels in HSC-T6, which has a lower capacity to remove cholesterol compared to hepatocytes. On the contrary, in AML-12, accumulation of FC in the presence of CA was not enhanced, indicating removal of cholesterol access. Hepatocytes possess multiple output mechanisms of cholesterol, exclusively metabolizing it into bile acids or directly extracting it through the ABCG5/G8 receptor [40], which may further contribute to the prevention of toxic accumulation of FC in hepatocytes. In addition, FC toxicity could be eliminated via cholesterol ester synthesis within hepatocytes [41]. In HSC, we demonstrated cholesterol-induced oxidative and mitochondrial stress. Corresponding to the enhanced FC accumulation, cholesterol treatment significantly elevated lipid peroxidation in HSC-T6 cells (200 µM CHOL + 200 µM CA). On the contrary, its effect on the hepatocytes, although significant, was weaker. Corresponding to this observation, cholesterol treatment in HSC showed a reduction in mitochondrial membrane potential, while cholic acid alone did not alter the mitochondrial membrane potential. On induction of mitochondrial leakage of cytochrome C, apoptosis was facilitated and mitochondrial outer membrane permeabilization usually led a cell to die. Corresponding to the cholesterol-mediated oxidative and mitochondrial stress in HSC, it also induced cytochrome c leakage, elevation of caspase-3 activity, and DNA fragmentation, indicating a pro-apoptotic effect of cholesterol in stellate cells.

Cholesterol levels administered in the presence of bile acids are doubled in the liver compared to the control and are significantly higher compared to administration of only cholesterol. We considered the inhibition of the FXR-cholesterol removal pathway by bile acids’ co-administration as the trigger for the inflammatory response, along with proliferation and induction of stellate cell apoptosis, all induced by cholesterol. Currently, the ratio and level of bile acids to free cholesterol, or how bile acids in addition to CA can facilitate the accumulation of cholesterol in the liver, are not known and need to be elucidated. It was indicated that a serum ratio of 2.7 of TBA to TC points to cirrhosis [31]. It could be considered that treatment for a short time with cholesterol in the presence of CA may inflict a revisable inflammatory/proliferation stimulus that is followed by HSC apoptosis and mitigation of fibrosis.

## 5. Conclusions

We used two mice models of bile acid toxicity with and without exposure to dietary cholesterol to induce liver inflammation and fibrosis. Moreover, we examined the effect of cholesterol and cholic acid in vitro on the apoptosis of activated HSC and hepatocytes. We showed that dietary cholesterol ameliorates bile acid toxicity and liver damage while activating the inflammatory/proliferative pathways. Cholesterol also ameliorates advanced fibrosis and induces HSC apoptosis. Furthermore, we demonstrated a toxic cholesterol effect in HSC cells by oxidative stress and mitochondrial damage. Cholesterol accumulation showed decreased HSC activation and induced oxidative and mitochondrial stress, and it further facilitated programmed cell death to eliminate HSC and mitigate liver fibrosis.

## Data Availability

Data is contained within the article and supplementary materials.

## Supplementary Materials

The following supporting information can be downloaded at: https://www.mdpi.com/article/10.3390/antiox11030536/s1, Figure S1: Effect of dietary cholesterol and cholic acid on weight gain and food intake, Figure S2: Effect of dietary cholesterol and cholic acid on body, adipose tissue and liver weight, Figure S3: Effect of dietary cholesterol and cholic acid on liver inflammatory response, Figure S4: Effect of dietary cholesterol and cholic acid on fasting blood glucose and serum lipids profile, Figure S5: Effect of cholesterol supplementation on liver αSMA Immunohistochemical staining in MDR2KO mice; Table S1: Antibodies; Table S2: Diet composition; Table S3: PCR primers; Tables S4–S8: Protocols for experiments.

## Abbreviations

CHOL	Cholesterol
CA	Cholic acid
Col α1[I]	pro-collagen α1(I)
Col α1[III]	pro-collagen α1(III)
ECM	Extracellular matrix
FC	Free cholesterol
GSH	Glutathione
HSC	Epatic stellate cells
MDA	Malondialdehyde
Mdr2	Multi-drug resistance 2
mGSH	Mitochondria glutathione
MMP-2	Matrix metalloproteinases 2
NAFLD	Nonalcoholic fatty liver disease
NASH	Nonalcoholic steatohepatitis
ND	Normal diet
ROS	Reactive oxygen species
ALT/SGPT	Serum alanine aminotransferase
AST/SGOT	Serum aspartate aminotransferase
TBA	Total bile acids
TIMP-2	Tissue inhibitors of metalloproteinases-2
TNFα	Tumor necrosis factor α
αSMA	α-smooth muscle actin
