# Supplementary material for: Cholesterol Induces Oxidative Stress, Mitochondrial Damage and Death in Hepatic Stellate Cells to Mitigate Liver Fibrosis in Mice Model of NASH
Source: Antioxidants (Basel). 2022 Mar 11;11(3):536. doi: 10.3390/antiox11030536 (PMC8944482; doi:10.3390/antiox11030536)
Supplement: Supplementary file 1 [file antioxidants-11-00536-s001.zip › antioxidants-1592285-supplementary.pdf]

**1.Supplamentry Results**

**1.1 Effect of dietary cholesterol and cholic acid on weight gain and food intake**

Mice weights were measured every three to four days during the 6 weeks of the study. As presented in Supplementary figure S1A, the cholesterol supplemented group (CHOL group) has shown similar weight gain during the experiment compared to the ND group (control group). Simultaneously, supplementation with cholic acid (CA and CHOL+CA groups) decreased mice weight gain significantly compared to ND. The trend continued throughout the experiment, including the day on which animals were sacrificed following 12 h of fasting (Supplementary figure S1B). Furthermore, Mice food intake was measured every three to four days during the 6 weeks of the study. Over experiment duration, no significant difference was observed between the experimental groups (Supplementary figure S1C).

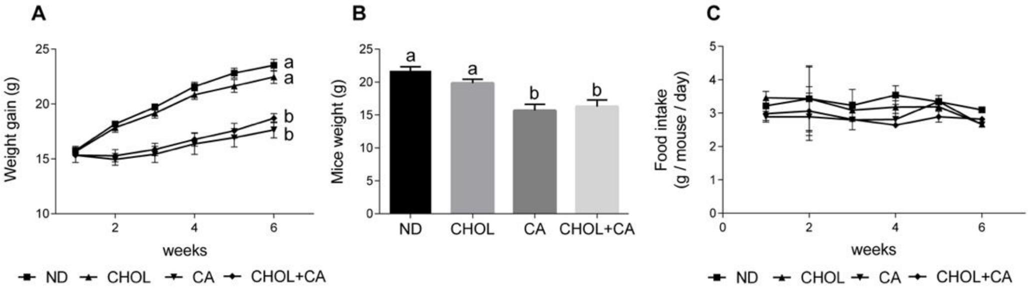


**Supplementary figure S1**. **Effect of dietary cholesterol and cholic acid on weight gain and food intake.** Male C57BL/6J mice aged 4-5 weeks were fed: ND-Normal diet (n=10), CHOL- Cholesterol diet (n=10), CA- Cholic acid diet (n=9), CHOL+CA-Cholesterol+ Cholic acid diet (n=8) for 6 weeks. **(A)** Body weight over experiment duration. **(B)** Average Body weight at sacrifice. **(C)** Food intake over experiment duration. All values are expressed as mean ± SEM. Columns indicated with different letters (a, b) are significantly different ($p<0.05$) in Tukey- Kramer post hoc test.

**1.2 Effect of dietary cholesterol and cholic acid on body, adipose tissue and liver weight**

Following the 6 weeks of feeding, mice livers were obtained and weighed after the sacrifice. As presented in Supplementary figure S2A, CHOL+CA group showed higher liver weights compared to ND, CHOL and CA groups ($p<0.0001$). Furthermore, CHOL+CA group showed increased liver to body weight ratio compared to all other experimental groups ($p<0.0001$), Supplementary figure S2B. Cholic acid supplementation (CA group) also elevated liver to body weight ratio significantly compared to ND, yet not as high as CHOL+CA

(Supplementary figure S2B). Mice epididymal adipose tissue (eWAT) was obtained and weighed after the sacrifice. While no significant difference of eWAT weight between the ND and CHOL group was observed, groups supplemented with cholic acid (CA and CHOL+CA) showed a significant decrease in eWAT weight compared to ND and CHOL (Supplementary figure S2C).

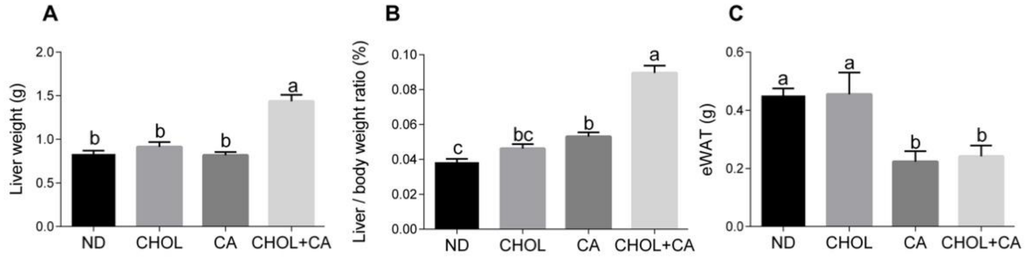


**Supplementary figure S2. Effect of dietary cholesterol and cholic acid on body, adipose tissue and liver weight.** Male C57BL/6J mice aged 4-5 weeks were fed: ND-Normal diet (n=10), CHOL- Cholesterol diet (n=10), CA- Cholic acid diet (n=9), CHOL+CA-Cholesterol+ Cholic acid diet (n=8) for 6 weeks. **(A)** Liver weight at sacrifice. **(B)** Liver to body weight ratio at sacrifice. **(C)** eWAT weight at sacrifice. All values are expressed as mean ± SEM. Columns indicated with different letters (a, b, c) are significantly different ($p<0.05$) in Tukey- Kramer post hoc test.

### 1.3 Effect of dietary cholesterol and cholic acid on liver inflammatory response

Our study evaluated the expression of multiple inflammatory markers following bile acid-induced damage with and without cholesterol. iNOS is a nitric oxide synthase expressed in response to inflammatory cytokines (1). As presented in Supplementary figure S3A, ND, CHOL, and CA groups expressed low mRNA level of iNOS while addition of cholesterol to cholic acid supplementation (CHOL+CA group) increased its expression significantly. The same effect of CHOL+CA diet was observed in iNOS protein expression, as shown in Supplementary figure S3B. SAA1 and SAA2 are vital acute phase response proteins in the liver(2),their expression levels were upregulated in CHOL+CA group ($p<0.001$), while the rest of the study groups (ND, CHOL and CA) manifested similarly low expression levels (Supplementary figure S3C and S3D). TNF-α, an indicator of general inflammatory response, and IL-6, a macrophage secreted cytokine which regulates immune response(3,4),were also upregulated in CHOL+CA group ($p<0.001$), while ND, CHOL and CA manifested similarly low expression levels (Supplementary figure S3E and S3F).

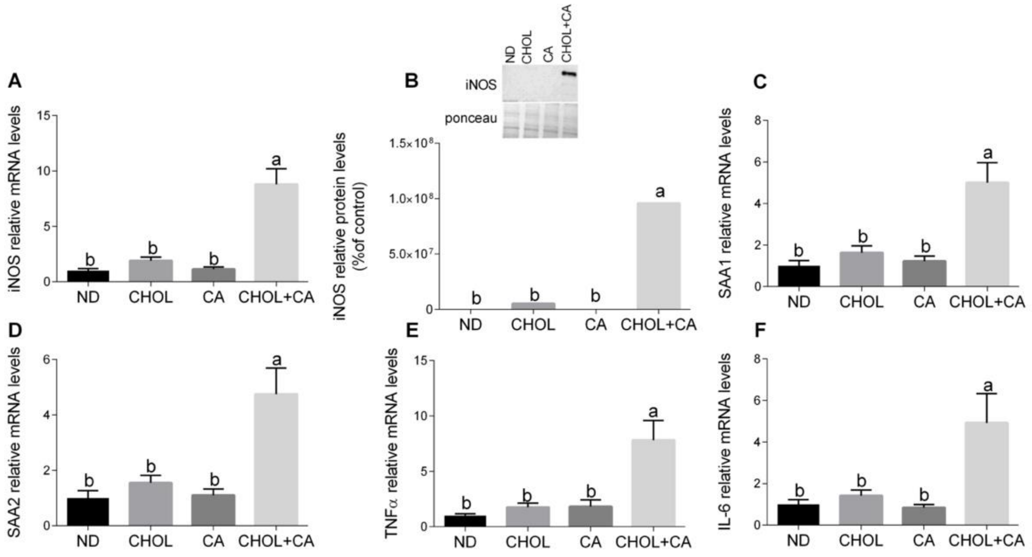


**Supplementary figure S3. Effect of dietary cholesterol and cholic acid on liver inflammatory response.** Male C57BL/6J mice aged 4-5 weeks were fed: ND-Normal diet (n=10), CHOL-Cholesterol diet (n=10), CA- Cholic acid diet (n=9), CHOL+CA-Cholesterol+ Cholic acid diet (n=8) for 6 weeks. **(A)** iNOS relative mRNA levels. **(B)** iNOS relative protein levels **(C)** SAA-1 relative mRNA levels. **(D)** SAA-2 relative mRNA levels. **(E)** TNF-α relative mRNA levels. **(F)** IL-6 relative mRNA levels. All values are expressed as mean ± SEM. Columns indicated with different letters (a, b) are significantly different ($p<0.001$) in Tukey- Kramer post hoc test.

**1.4 Effect of dietary cholesterol and cholic acid on fasting blood glucose, serum lipids profile and liver free cholesterol content.**

As presented in Supplementary figure S4A, it was found that there were no significant differences in fasting blood glucose levels between all experimental groups. As presented in Supplementary figure S4B, serum total cholesterol levels were significantly elevated in the CHOL+CA group compared to all the other study groups. Serum triglycerides level, presented in Supplementary figure S4C, were significantly decreased in CHOL and CA groups,

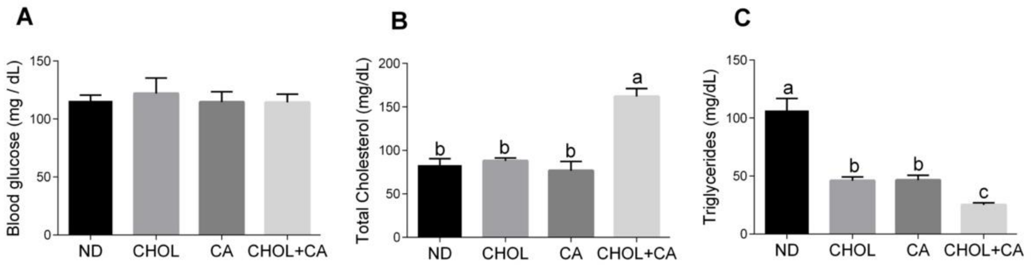

compared to ND. Additionally, in the CHOL+CA group, TG levels in serum were significantly lower than ND, CHOL, and CA.

**Supplementary figure S4. Effect of dietary cholesterol and cholic acid on fasting blood glucose and serum lipids profile**. Male C57BL/6J mice aged 4-5 weeks were fed: ND-Normal diet (n=10), CHOL- Cholesterol diet (n=10), CA- Cholic acid diet (n=9), CHOL+CA-Cholesterol+ Cholic acid diet (n=8) for 6 weeks. (A) Fasting blood glucose level. (B) Serum cholesterol level (C) Serum triglycerides level. All values are expressed as mean ± SEM. Columns indicated with different letters (a, b, c) are significantly different ($p<0.05$) in Tukey-Kramer post hoc test.

**1.5 Cholesterol supplementation eliminates hepatic stellate cells in MDR2(-/-) mice.**

As presented in Supplementary figure S5A, cholesterol supplementation decreased α-SMA positive cells presence IN Mdr2 (-/-) mice. Further investigation revealed that cholesterol supplementation (CHOL group) in Mdr2 (-/-) mice decreased significantly α-SMA protein expression compared to ND group (Supplementary figure S5B).

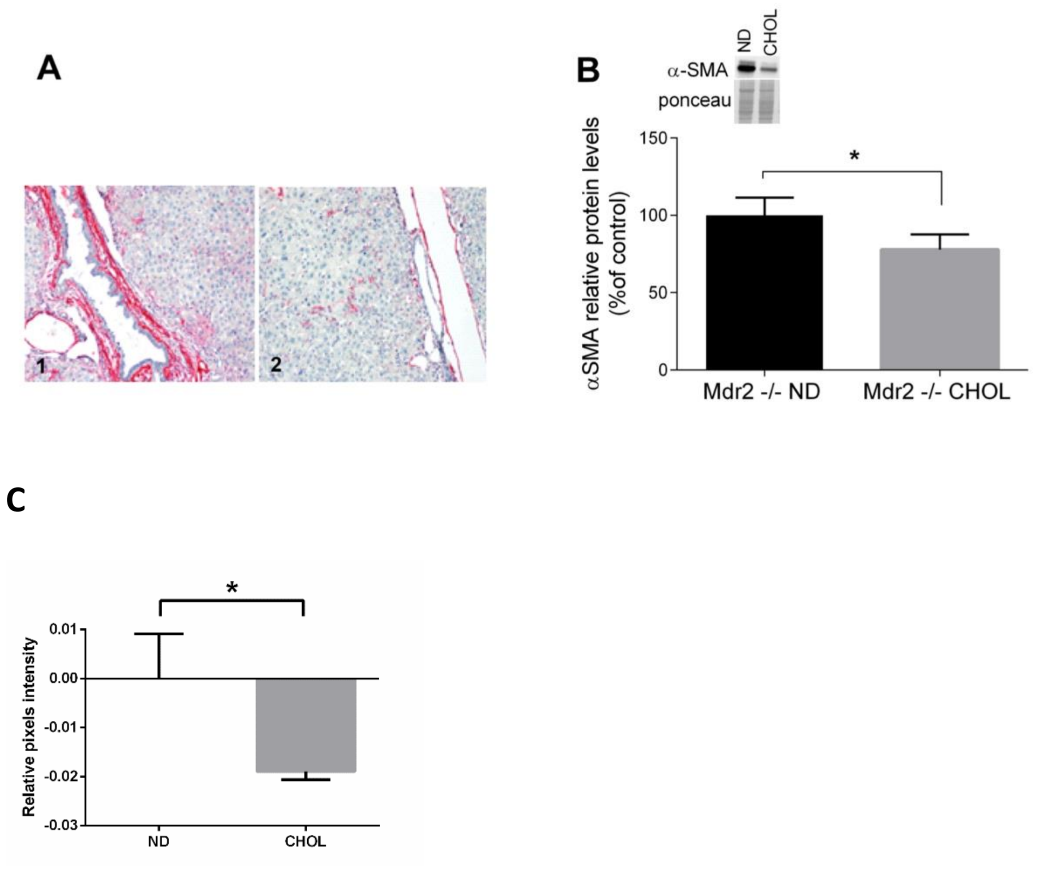

**Supplementary figure S5.** Effect of cholesterol supplementation on liver αSMA Immunohistochemical staining in MDR2KO mice**. (A)** Representative histology of liver αSMA Immunohistochemical staining, X10 magnification, ND (1) and CHOL (2). **(B)** α-SMA relative protein levels. **(C)** Histological sections of three individuals from each group were imaged utilizing EVOS M500 microscope. Two frames from each slide were captured at magnification of X200. All captured images were at resolution of 2048 x 1536 pixels and saved as tiff files. ImageJ software was employed to determine the relative intensity of pixels from Red channel (Red/Green+Blue) of the total area of each image. For graphical presentation the average value of relative pixels intensity of ND group was subtracted from those of all treatments. All values are expressed as mean ± SEM, columns marked with * are different from Control according to Student t-test ($p < 0.05$).

## Methods

**Protein extraction and Western blot analysis.** Protein extraction and Western blot analysis were performed as described previously. In short, mouse liver tissue or cultured HCS-T6 and AML-12 on 6 well plate, were lysed with a buffer containing 20mM Tris–HCl (pH7.5), 145mM NaCl, 10% Glycerol, 5mM EDTA, 0.1% Triton X-100, 100mM PMSF,100mM $NaVO_4$, 5mM NaF, and 1% protease inhibitor cocktail. Protein concentrations were determined using BSA standard curve and Bradford assay. Equal amounts of protein lysates were separated by SDS–PAGE, transferred to nitrocellulose membranes, blotted with primary antibodies overnight at 4 °C and Peroxidase-conjugated Affini-Pure Goat Anti-Mouse or Anti-Rabbit IgG secondary antibody (Jackson Immuno Research Laboratories, Inc.) for 1h at room temperature. The band optical density was analyzed on an Image lab system (Bio-Rad) and normalized to housekeeping protein or to lane total protein content visualized with Ponceau S solution (Sigma-Aldrich, USA). All antibodies are described in Supplementary Table S1.

**Quantitative real-time polymerase chain reaction.** Total RNA was isolated from liver tissue or tissue or cultured HCS-T6 cultured on 6-well plate, using Tri-Reagent (Sigma–Aldrich, Rehovot, Israel), according to the manufacturer's protocol. Complementary DNA was prepared with a High-Capacity cDNA Reverse Transcription Kit (Quanta BioSciences, Gaithersburg, MD, USA). Quantitative real-time PCR (RT-qPCR) was performed with the 7300 Real-Time PCR System (Applied Biosystems, Foster City, CA, USA) with specific primers (Table 2). Quantitative changes in gene expression were determined by normalizing to 18S mRNA. All primers are described in Supplementary Table S3.

**Supplementary Table S1: Antibodies**

| Antibody | company | dilution |
|---|---|---|
| α -Smooth Muscle Actin (immunohistochemical assay) | Novus bio | 1:1600 |
| α -Smooth Muscle Actin (western blot analysis) | Abcam, Plc, UK | 1:1000 |
| Cleaved caspase-3 (immunohistochemical assay) | Abcam, Plc, UK | 1:1000 |
| iNOS | BD-Biosciences, Inc, USA | 1:1000 |
| Cytochrome c | Santa Cruz Biotechnology | 1:1000 |
| Peroxidase-conjugated Affini-Pure Goat Anti-Mouse IgG secondary antibody. | Jackson Immuno Research Laboratories, Inc, USA | 1:10,000 |
| Peroxidase-conjugated Affini-Pure Goat Anti-Rabbit IgG secondary antibody. | Jackson Immuno Research Laboratories, Inc, USA | 1:10,000 |

**Supplementary table S2: Diet composition**

| | ND group | | CHOL group | | CA group | | CHOL+CA group | |
|---|---|---|---|---|---|---|---|---|
| | AIN-93 | | AIN-93 + 1 % (w/w) Cholesterol | | AIN-93 + 0.5 % (w/w) Cholic acid | | AIN-93 + 1 % (w/w) Cholesterol + 0.5 % (w/w) Cholic acid | |
| | g / kg | kcal / kg | g / kg | kcal / kg | g / kg | kcal / kg | g / kg | kcal / kg |
| Corn starch | 397.5 | 1590 | 397.5 | 1590 | 397.5 | 1590 | 397.5 | 1590 |
| Maltodextrin | 132 | 528 | 132 | 528 | 132 | 528 | 132 | 528 |
| Sucrose | 100 | 400 | 100 | 400 | 100 | 400 | 100 | 400 |
| Casein | 200 | 800 | 200 | 800 | 200 | 800 | 200 | 800 |
| Soybean oil | 70 | 630 | 70 | 630 | 70 | 630 | 70 | 630 |
| Cellulose | 50 | --- | 50 | --- | 50 | --- | 50 | --- |
| Mineral mix (AIN-76, S10022M) | 35 | --- | 35 | --- | 35 | --- | 35 | --- |
| Vitamin mix (AIN-93VX, V10037) | 10 | 40 | 10 | 40 | 10 | 40 | 10 | 40 |
| L-Methionine | 3 | 12 | 3 | 12 | 3 | 12 | 3 | 12 |
| Choline chloride | 2.5 | --- | 2.5 | --- | 2.5 | --- | 2.5 | --- |
| BHT | 0.014 | --- | 0.014 | --- | 0.014 | --- | 0.014 | --- |
| Cholesterol | 0 | --- | 10 | --- | 0 | --- | 10 | --- |

| | | | | | | | | |
|---|---|---|---|---|---|---|---|---|
| Cholic acid | 0 | --- | 0 | --- | 5 | --- | 5 | --- |
| | | | | | | | | |

**Supporting Table S3 (PCR primers )**

| **Mouse** | **Forward** | **Reverse** |
|---|---|---|
| iNOS | 5’- AGCTCCCTCCTTCTCCTTCT -3' | 5’- TCTCTGCTCTCAGCTCCAAG-3’ |
| TNF-α | 5’- ACGTGGAACTGGCAGAAGAG -3’ | 5’- CCACAAGCAGGAATGAGAAGA -3’ |
| SAA-1 | 5’- GATGAAGCTACTCACCAGCCT -3’ | 5’- GGTCAGCAATGGTGTCCTCA -3’ |
| SAA-2 | 5’- AATACTTCCCATGCTCGGGGG -3’ | 5’- TTTTCTCAGCAGCCCAGACT -3’ |
| TGF-β1 | 5’-GCGGACTACTATGCTAAAGAGG -3’ | 5’-GTAGAGTTCCACATGTTGCTCC-3’ |
| PDGFA | 5’-GAGATACCCCGGGAGTTGAT-3’ | 5’-AAATGACCGTCCTGGTCTTG-3’ |
| PDGFB | 5’-GGGTGGGACTTTGGTGTAGAGAGG3’ | 5’-TTGAGGCGAGAATGCC-3’ |
| PDGF-R | 5’TCAACGACTCACCAGTGCTC--3’ | 5’-TTCAGAGGCAGGTAGGTGCT-3’ |
| Cola1 (I) | 5’-AGGGCGAGTGCTGTGCTTT-3’ | 5’-CCCTCGACTCCTACATCTTCTGA-3’ |
| Cola1 (III) | 5’-TGAAACCCCAGCAAAACAAAA-3’ | 5’-TCACTTGCACTGGTTGATAAGATTAA-3’ |
| αSMA | 5’-AAACAGGAATACGACGAA-3’ | 5’-CAGGAATGATTTGGAAAGGA-3’ |
| MMP-2 | 5'-CACCTGGTTTCACCCTTTCTG-3' | 5'-AACGAGCGAAGGGCATACAA-3' |
| TIMP-2 | 5’-TATCTACACGGCCCCCTCTT-3’ | 5’-TCCCAGGGCACAATGAAGTC-3’ |
| CTGF | 5’-CTGCCAGTGGAGTTCAAATGC-3’ | 5’-TCATTGTCCCCAGGACAGTTG-3’ |
| FGF- β | 5’-AGCGACCCACACGTCAAACTA-3’ | 5’-CAGCCGTCCATCTTCCTTCATA-3’ |
| 18s | 5’-AAACGGCTACCACATCCAAG -3’ | 5’- CCTCCAATGGATCCTCGTTA -3’ |
| **Rat** | **Forward** | **Reverse** |
| αSMA | 5’-ACAACGTGCCTATCTATGAGGGCT-3’ | 5’-AGCGACATAGCACAGCTTCTCCTT-3’ |
| Cola1 | 5’-CCGTGACCTCAAGATGTGCC-3’ | 5’-GCTCATACCTTCGCTTCCAA-3’ |
| MMP-2 | 5’-GGTGACCTTGACCAGAAC-3’ | 5’-GTTACGTCGCTCCATACT-3’ |
| MMP-9 | 5’-CAATCCTTGCAATGTGGATG-3’ | 5’-CTGCGGATCCTCAAAGGC-3’ |
| c-Myc | 5’-GAAACGGCGAGAACAGTTGA-3’ | 5’-CCAAGGTTGTGA GTTGAGCAGC -3’ |
| 18s | 5’-CACGGACAGGATTGACAGAT -3’ | 5’- CAAATCGCTCCACCAACTAA -3’ |

Supporting Table S**4**

| Lysis Buffer | Final Concentration |
|---|---|
| Trizma Base (pH 7.4) | 20 mM |
| NaCl | 145 mM |
| Glycerol | 10 % |
| EDTA | 5 mM |
| Triton X-100 | 1 % |
| Phenylmethylsulphonyl fluoride (PMSF) | 100 mM |
| $NaVO_4$ | 200 mM |
| NaF | 5 mM |
| Protease inhibitors cocktail | 1 % |

Supporting Table S**5**

| Lysis buffer -cytosolic fraction | Final Concentration |
|---|---|
| NaCl | 150 mM |
| HEPES (pH 7.4) | 50 mM |
| Digitonin | 25 µg/mL |
| Hexylene glycol | 1 M |
| Protease inhibitors cocktail | 1% (v/v) |

Supporting Table S**6**

| Lysis buffer - membrane-bound organelles fraction | Final Concentration |
|---|---|
| NaCl | 150 mM |
| HEPES (pH 7.4) | 50 mM |
| NP-40 | 1% (v/v) |
| Hexylene glycol | 1 M |
| Protease inhibitors cocktail | 1% (v/v) |

Supporting Table S7

| Sample Buffer X3 | |
|---|---|
| Substance | Final Volume |
| Trizma base 1.5 M, pH 8.8 | 2.5 mL |
| SDS 10% | 4 mL |
| Glycerol | 2 mL |

| | |
|---|---|
| β - mercaptoethanol | 1 mL |
| Bromophenol blue 0.5 % | 400 µL |
| DDW | Complete to 10 ml |
| Sample Buffer X6 | |
| Substance | Final Volume |
| Tris HCl | 15 ml |
| SDS powder | 6 gr |
| Glycerol 100% | 20 ml |
| DDT | 1.1565 g |
| Bromophenol blue 0.5 % | 400 µL |
| DDW | Complete to 50 ml |

Supporting table S**8**

| DNAF staining solution | Final volume / weight |
|---|---|
| PI | 5 mg |
| Triton X-100 | 100 µL |
| Sodium citrate | 100 mg |
| DDW | Up to 100 mL |

References


1. Chen T, Zamora R, Zuckerbraun B, Billiar TR. Role of Nitric Oxide in Liver Injury. Current Molecular Medicine. 2003 Sep 1;3(6):519–26.
2. Thorn CF, Lu Z-Y, Whitehead AS. Regulation of the Human Acute Phase Serum Amyloid A Genes by Tumour Necrosis Factor-alpha, Interleukin-6 and Glucocorticoids in Hepatic and Epithelial Cell Lines. Scandinavian Journal of Immunology. 2004 Feb;59(2):152–8.
3. Tekkesin N, Taga Y, Sav A, Almaata I, Ibrisim D. Induction of HGF and VEGF in hepatic regeneration after hepatotoxin-induced cirrhosis in mice. Hepato-gastroenterology. 58(107–108):971–9.

4. Koyama Y, Brenner DA. Liver inflammation and fibrosis. Journal of Clinical Investigation. 2017 Jan 3;127(1):55–64.
